# Hypoxia-induced PTTG3P contributes to colorectal cancer glycolysis and M2 phenotype of macrophage

**DOI:** 10.1042/BSR20210764

**Published:** 2021-07-06

**Authors:** Yue Wang, Guilin Yu, Yiyang Liu, Longfei Xie, Jinnian Ge, Guohua Zhao, Jie Lin

**Affiliations:** 1 Department of General Surgery, Cancer Hospital of China Medical University, Liaoning Cancer Hospital and Institute, Liaoning 110042, P.R. China; 2 Department of Surgery, Affiliated Hospital of Youjiang Medical University for Nationalities, 533000, Guangxi Zhuang Autonomous Region, P.R. China; 3 Department of Physics and Integrative Biology, University of California, Berkeley, CA 94720, U.S.A.; 4 Department of General Surgery, The Central Hospital of Shenyang Medical College, Liaoning 110031, P.R. China

**Keywords:** hypoxia, pseudogene, PTTG3P, YAP1

## Abstract

Long noncoding RNAs (lncRNAs) play critical factors in tumor progression and are ectopically expressed in malignant tumors. Until now, lncRNA pituitary tumor-transforming 3, pseudogene (PTTG3P) biological function in colorectal cancer (CRC) further needs to be clarified. qRT-PCR was used to measure the PTTG3P level and CCK-8, glucose uptake, lactate assay, adenosine triphosphate (ATP) assay, extracellular acidification rate (ECAR) assay, and xenograft mice model were adopted to evaluate the glycolysis and proliferation, and macrophage polarization were determined in CRC cells. Xenograft experiments were utilized to analyze tumor growth. Ectopic expression of PTTG3P was involved in CRC and related to dismal prognosis. Through gain- and loss-of-function approaches, PTTG3P enhanced cell proliferation and glycolysis through YAP1. Further, LDHA knockdown or glycolysis inhibitor (2-deoxyglucose (2-DG), 3-BG) recovered from PTTG3P-induced proliferation. And PTTG3P overexpression could facilitate M2 polarization of macrophages. Silenced PTTG3P decreased the level of inflammatory cytokines TNF-α, IL-1β and IL-6, and low PTTG3P expression related with CD8^+^ T, NK, and TFH cell infiltration. Besides, hypoxia-inducible factor-1α (HIF1A) could increase PTTG3P expression by binding to the PTTG3P promoter region. Hypoxia-induced PTTG3P contributes to glycolysis and M2 phenotype of macrophage, which proposes a novel approach for clinical treatment.

## Introduction

Colorectal cancer (CRC) remains one of the major triggers of deaths from malignant tumors. Globally more than 1 million people suffer from CRC every year [1]. As of 2012, CRC is the fourth cause of cancer deaths after lung, stomach, and liver cancer [2]. Despite improvements in diagnosis and combined treatment, patients with CRC have an even worse prognosis, especially in advanced patients. Therefore, it is quite urgent to clarify the mechanism, even potential approaches for the therapeutic intervention of CRC.

Accumulating evidence has shown that pseudogene, a type of long noncoding RNA (lncRNA), exhibits pivotal functions. It is estimated that the human genome has more than 18000 pseudogenes [3]. And pseudogene has emerged as key regulators of important biological processes involved in the development of human cancers. For instance, increased CYP4Z1 expression promotes tumor angiogenesis and growth in breast cancer partly via PI3K/Akt and ERK1/2 activation [4]. Nanog regulates primitive hematopoiesis by directly repressing critical erythroid lineage specifiers [5]. PTENP1 can exert a growth-suppressive role by regulating cellular levels of PTEN [6]. NAIL identified in colitis patients that regulates inflammation through NFkB which is important for the inflammation [7], and cross-talk of NFkB and HIF signaling pathways in mediating the occurrence of inflammation. LncRNA PTTG3P (pituitary tumor-transforming 3, pseudogene, NR_002734), located at chromosome 8q13.1, was first reported in the study of the human pituitary tumor transforming gene (*hPTTG*) family in 2000 [8]. PTTG3P plays a vital role in CRC, Liu et al. [9] revealed that lncRNA PTTG3P was up-regulated in CRC cancer tissues by lncRNA databases, and could function as an indicator for the prognosis in CRC. However, its biological function in M2 phenotype of macrophage is yet to be illustrated in CRC.

Our data discovered that PTTG3P predicts poor prognosis in patients with CRC. Further study revealed that PTTG3P facilitates cell growth by regulating hypoxia-inducible factor-1α (HIF1A)/PTTG3P/YAP1 axis, and induces M2 polarization of macrophages.

## Materials and methods

### Clinical samples

A total of 120 patients with CRC were enrolled from the Affiliated Hospital of Youjiang Medical University for nationalities, the Central Hospital of Shenyang Medical Hospital, and Cancer Hospital of China Medical University between March 2010 and November 2015. The including criteria were as follows: patients with a definite pathological diagnosis; no patients were received with chemotherapy or radiotherapy before surgery, and the characteristics of cases were thoroughly noted. The tumor and paired non-tumor tissues were also collected after lesion excision with 30 min and stored in liquid nitrogen, then transferred to a −80°C refrigerator. And the characteristics of cases were thoroughly noted. All the CRC patients have signed informed consent before utilizing the clinical resources for investigation aims. The study was approved by the Ethics Committee of Youjiang Medical University for nationalities and Cancer Hospital of China Medical University.

### Cell lines culture

Five human CRC cell lines (HT-29, SW620, HCT-8, SW480, and HCT116) and normal human intestinal epithelial cell lines (FHC, NCM460) were obtained from ATCC (Manassas, VA, U.S.A.). And cultured according to their instructions. All cells were cultured in an incubator according to their instructions at 37°C and in a humidified atmosphere with 5% CO_2_.

### Total RNA isolation, qRT-PCR, and transfection

The expression levels of RNA were calculated by the qRT-PCR system. Total RNA was extracted by TRIzol Reagent (Invitrogen), and 1 μg of total RNA was reverse transcribed using the PrimeScript RT Reagent Kit (Perfect Real-Time; Takara). pcDNA3.1-PTTG3P, PTTG3P-containing lentiviral sequence vector (sh-PTTG3P) were purchased from GeneChem Corporation (Shanghai, China). CRC cells were transfected with plasmids in the presence of Lipofectamine 3000 (Invitrogen). After 48 h of transfection, cells were gathered for further use in the following experiments. The gene expression quantity was calculated using the 2^−ΔΔ*C*_t_^ method. The oligonucleotides were transfected into CRC cells using Lipofectamine 2000 (Invitrogen, U.S.A.) and the transfection efficiency was confirmed by qRT-PCR. The details are listed in Supplementary Tables S1 and S2.

### Cell proliferation assay

Cell viability assay was carried out to analyze cell proliferation. Cell viability was estimated using CCK8 (CK04, DOJINDO, Beijing, China), based on the manufacturer’s instructions. Cells were seeded in 96-well culture plates. After incubation for the indicated time, a CCK-8 reagent (10 μl) was added to each well. Cell viability was measured with a microplate reader for the absorbance at a wavelength of 450 nm.

### Flow cytometry of apoptosis

CRC cells in six-well plates were rinsed in phosphate buffer saline (PBS), and then were trypsinized and resuspended in 100 μl binding buffer added with 2.5 μl of fluorescein isothiocyanate (FITC) conjugated Annexin V and 1 μl of PI (Invitrogen). Fifteen minutes later, flow cytometry (BD Biosciences) was utilized for apoptotic cells.

### Glucose, lactate, adenosine triphosphate levels, and extracellular acidification rate

Glucose, lactate, adenosine triphosphate (ATP) levels, and extracellular acidification rate (ECAR). The levels of glucose and lactate were calculated with a Glucose Colorimetric Assay Kit (BioVision, CA) and a Lactate Assay Kit (BioVision, CA) in line with the instructions of the manufacturer. ATP level was tested using Cell Titer-Glo Luminescent Cell Viability Assay (Promega, Madison, MI). ECAR was detected using Seahorse XF 96 Extracellular Flux Analyzer (Agilent Technologies, Santa Clara, CA) according to the manufacturer’s instructions.

### Chromatin immunoprecipitation assay

CRC cells (2 × 10^6^) were used for chromatin immunoprecipitation (ChIP) assay according to the manufacturer’s protocol of hIP)assay kit (Millipore). The resulting precipitated DNA specimens were analyzed using PCR to amplify fractions of the PTTG3P promoter. The PCR products were resolved electrophoretically on a 2% agarose gel and visualized using Ethidium Bromide staining.

### Animal study

HCT116 cells were transfected with sh-PTTG3P. A total of 1 × 10^7^ indicated cells were subcutaneously injected into 4-week-old male nude mice. Tumor volume measured every 5 days. Isoflurane gas anesthesia was used in mice. 2-Deoxyglucose (2-DG) using in the study was injected into the abdominal cavity (1000 mg/kg, injected into the abdominal cavity). Oxaliplatin using in the study was injected into the abdominal cavity (5 mg/kg twice per week). After 35 days, the 20 mice were sacrificed by excessive carbon dioxide asphyxiation euthanasia, and the tumor weight was measured. The animal study was carried out in Youjiang Medical University following the Guide for the Care and Use of Laboratory Animals of the NIH. The present study had been approved by the Committee on the Ethics of Youjiang Medical University and China Medical University.

### Statistical analysis

All the data were shown as the mean ± standard deviation, at least three independent experiments. The difference between two independent groups was analyzed by a two-tailed Student’s *t* test, while multigroup comparison was made by ANOVA. Expression correlation between genes was analyzed by Pearson correlation analysis. Survival analysis was conducted using the Kaplan–Meier method and analyzed by the log-rank test. SPSS 22.0 (SPSS Inc., Chicago, IL, U.S.A.) was used to conduct statistical analyses, and differences were ensured when *P*-value was <0.05.

## Results

### PTTG3P is highly expressed in CRC

To evaluate potential lncRNAs involved in mediating CRC progression, we examined the lncRNA expression profile (GSE 84983) (Supplementary Figure S1A). Comparison between CRC tumor tissues and adjacent normal tissues, we focused on the up-regulated lncRNAs (fold change > 5, *P*<0.01), for these lncRNAs might be oncogenes and therapeutic targets. LncRNA PTTG3P was one of the most up-regulated and chosen for consideration (Supplementary Figure S1B). Then, we found that PTTG3P had rarely the ability to code proteins, using the open-reading frames (ORFs) Finder and conserved domain database. Moreover, five other different online metrics got the same conclusion (Supplementary Table S3). Additionally, we identified no valid Kozak consensus sequence in PTTG3P [10], indicating that PTTG3P was an lncRNA with no protein-coding potential. Then we explored the subcellular location of PTTG3P by using lncRNA subcellular localization predictor software (lncLocator, http://www.csbio.sjtu.edu.cn/bioinf/lncLocator/) (Supplementary Figure S1C), suggesting PTTG3P was mainly localized to the cytoplasm, and subcellular fractionation confirmed the prediction (Supplementary Figure S1D).

**Figure 1:**
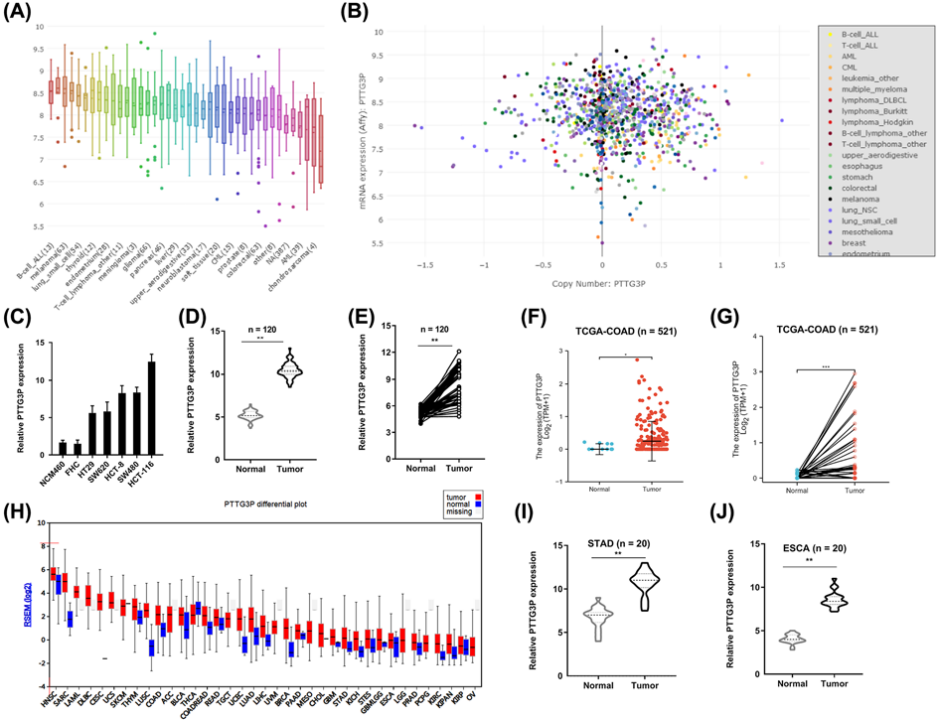
PTTG3P is up-regulated in CRC tissues and cell lines (**A,B**) Exploring PTTG3P expression in CRC cell lines by assembling the CCLE (www.broadinstitute.org/ccle). (**C**) The expression profiles of PTTG3P in HT-29, SW620, HCT-8, SW480, HCT116, NCM460 and FHC was detected with qRT-PCR. (**D,E**) qRT-PCR analysis was used to evaluate PTTG3P expression in 120 paired tumor and paired adjacent non-tumor tissues. (**F,G**) High PTTG3P expression was observed in the TCGA database of CRC (*n*=521). (**H**) High PTTG3P expression was observed in the TCGA database of malignant tumors. (**I,J**) High PTTG3P expression was observed in STAD and ESCA (*n*=20). *P<0.05, **P<0.01, ***P<0.001.

To verify the elevation of PTTG3P in CRC, we investigated the detailed annotative process of preclinical human cancer models via the Cancer Cell Line Encyclopedia (CCLE) (https://portals.broadinstitute.org/ccle), indicating that PTTG3P was remarkably overexpressed in cell lines of CRC (Figure 1A,B). Then, the cell lines of HT-29, SW620, HCT-8, SW480, HCT116, NCM460, and FHC were conducted for PTTG3P expression. As shown in Figure 1C, the PTTG3P expression was exceedingly increased in HT-29, SW620, HCT-8, SW480, HCT116 cells, compared with NCM460 and FHC cells.

Further, we explored PTTG3P expression in a cohort of 120 paired and non-tumor tissues of CRC, the clinicopathologic characteristics are demonstrated in Table 1. Significantly, the PTTG3P level was overexpressed in CRC tissues compared with their counterparts (Figure 1D,E), which was in accordance with the results of The Cancer Genome Atlas (TCGA) database (Figure 1F,G). Besides, high PTTG3P expression was observed in other malignant tumors (Figure 1H). Also, our specimens confirmed PTTG3P overexpression in stomach adenocarcinoma (STAD) and esophageal squamous cell carcinoma (ESCA) (Figure 1I,J). Intriguingly, there was no actionable EGFR, VEGFR or RAS mutations, indicating that higher expressed PTTG3P may be driven by oncogenic event (Supplementary Figure S1E–G). Altogether, these data revealed that PTTG3P was elevated in CRC and might be an oncogene.

**Table 1:** Correlation between PTTG3P expression and clinicopathologic characteristics of ovarian cancer patients

Variable	PTTG3P expression			*P*-value
	Total (*n*=120)	High expression	Low expression	
**Age (years)**				
≤60	52	27	26	
>60	68	32	35	0.86
**Gender**				
Male	56	30	28	
Female	64	29	33	0.74
**Tumor size (cm)**				
≤5	81	47	37	
>5	39	16	24	0.02
**Tumor invasion depth**				
T1–2	95	53	43	
T3–4	25	12	20	0.28
**Lymph node metastasis**				
N0	40	25	20	
N1–2	80	36	39	0.09
**Vessel invasion**				
Yes	65	49	20	
No	55	20	31	0.06
**Differentiation**				
Well	38	20	18	
Moderate	62	46	16	
Poor	20	13	7	0.01

### High PTTG3P level correlates with poor prognosis

**Figure 2:**
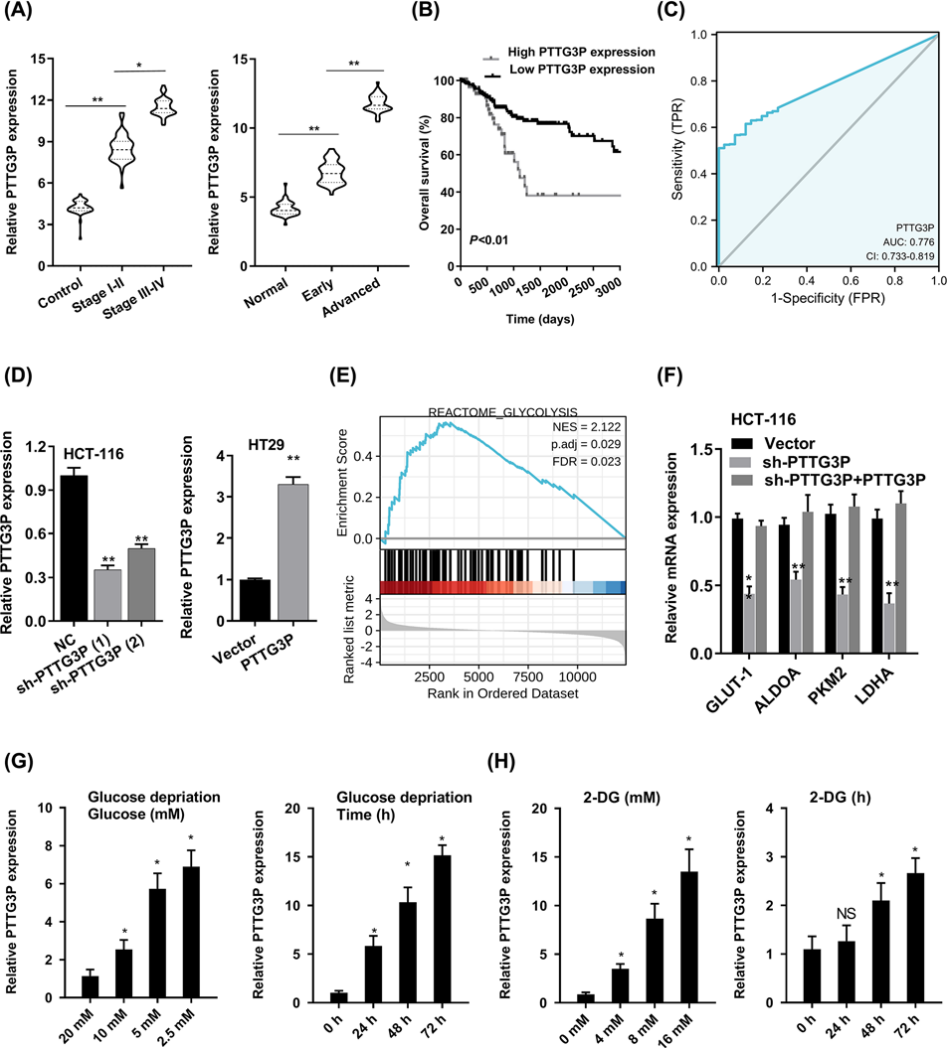
Overexpressed PTTG3P correlates with poor prognosis in CRC (**A**) The expression of PTTG3P was checked in different clinical stages of CRC tissues. (**B**) PTTG3P expression and survival predicted poor prognosis of OS in a cohort of 120 paired cases. (**C**) ROC curve of PTTG3P. (**D**) Short hairpin RNA (shRNA) targeting PTTG3P and PTTG3P overexpressed plasmids were transfected into HCT116 and HT-29 cells. (**E**) GSEA plot showing that PTTG3P expression positively correlated with glycolysis-activated gene signatures (REACTOME GLYCOLYSIS). (**F**) Analysis of glycolic gene expression in PTTG3P knockdown or re-expressed PTTG3P. (**G**) qPCR analysis showed that PTTG3P was up-regulated under low glucose culture conditions compared with normal glucose in a dose- and time-dependent manner. (**H**) qPCR analysis showed that PTTG3P was increased after 2-DG treatment in dose- and time-dependent manner. *P<0.05, **P<0.01.

To identify the connection between the level of PTTG3P and clinicopathologic features, we divided the cases into PTTG3P low- and high-expression groups based on the median expression. Up-regulated PTTG3P was positively linked with tumor size (*P*=0.02) and differentiation (*P*=0.01), but not with age (*P*=0.86), gender (*P*=0.74), tumor invasion depth (*P*=0.28), lymph node metastasis (*P*=0.09) or vessel invasion (*P*=0.06) (Table 1). Moreover, the PTTG3P expression in stage III–IV was higher than that in stage I–II tissues, and PTTG3P was expressed much more highly in advanced CRC samples than early CRC tissue (Figure 2A). Additionally, Kaplan–Meier survival curves illustrated that patients with highly expressed PTTG3P had poorer survival time (Figure 2B). Further, we determined the prognostic ability of PTTG3P in CRC. As shown in Table 2, univariate analyses suggested highly expressed PTTG3P was associated with a dramatic risk of death (*P*<0.01). Multivariate analysis demonstrated that PTTG3P expression was an independent prognostic factor (*P*<0.01). A model that incorporated the independent predictor was proposed as the nomogram (Supplementary Figure S2). Subsequently, the ROC curve was carried out to evaluate the diagnostic capacity of PTTG3P in CRC tissues compared with normal counterparts, the area under the ROC curve (AUC) was 0.776 (95% CI: 0.733–0.819) (Figure 2C). Thus, these data suggested that high expression of PTTG3P predicted a worse prognosis and may serve as a clinical biomarker for CRC patients.

**Table 2:** Univariate and multivariate analyses of clinicopathologic characteristics for correlations with overall survival

Variables	Univariate analysis		Multivariate analysis	
	HR (95% CI)	*P*-value	HR (95% CI)	*P*-value
PTTG3P expression	1.758 (1.085–2.850)	<0.01	1.712 (1.053–2.782)	<0.01
Tumor size	1.650 (1.086–2.508)	<0.01	1.923 (1.276–2.898)	<0.01
Differentiation	1.724 (1.183–2.511)	<0.01	1.724 (1.183–2.511)	<0.01

### PTTG3P is caused by metabolic stress and promotes glycolysis and proliferation in CRC

To investigate the biological function of PTTG3P, we transfected the PTTG3P overexpressed plasmids and shRNA targeting PTTG3P into HT-29 and HCT116 cells, respectively (Figure 2D). By determining PTTG3P expression via gene set enrichment analysis (GSEA), TCGA profiles, we found that PTTG3P level was positively correlated with the glycolysis by affecting genes in glycolysis regulation (Figure 2E). PTTG3P knockdown restrained the mRNA level of GLUT-1, ALDOA, PKM2 and LDHA, and the effect of sh-PTTG3P on glycolytic gene transcription could be rescued by PTTG3P re-expression (Figure 2F).

And glucose deprivation is a well-known feature of solid tumors. Subsequently, we wonder whether PTTG3P participated in cell survival under metabolic stress, then we carried out several experiments with different glucose concentration and glycolysis inhibitor 2-DG to make a condition of glucose deprivation. Obviously, PTTG3P expression was increased by glucose deprivation or 2-DG treatment in either dose- or time-dependent manner (Figure 2G,H). Thus, we elucidated that PTTG3P could play a crucial role in the progression of metabolic stress.

**Figure 3:**
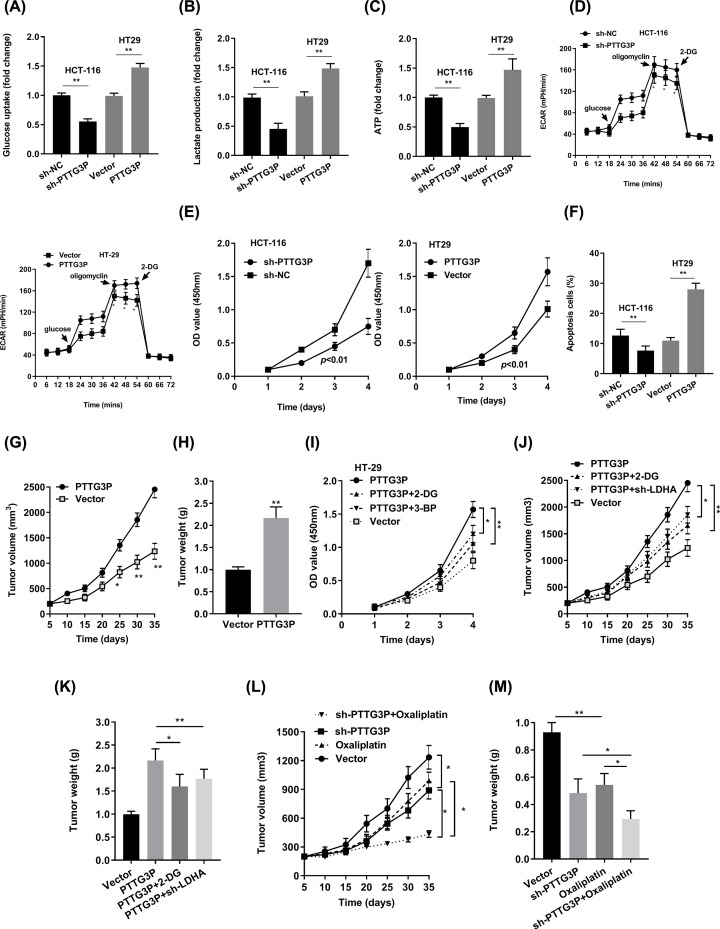
PTTG3P promotes the glycolysis and proliferation of CRC (**A**) Glucose uptake analysis. (**B**) Lactate production analysis. (**C**) ATP analysis explored the glucose uptake, lactate production, and ATP accumulation level, with sh-PTTG3P or overexpressed PTTG3P in HCT116 or HT-29 cells. (**D**) ECAR analysis tested the glycolytic capacity in CRC cells with sh-PTTG3P or overexpressed PTTG3P in HCT116 or HT-29 cells. (**E**) CCK8 assay detected the proliferation of HCT116 and HT-29 cells transfected with sh-PTTG3P or overexpressed PTTG3P. (**F**) Flow cytometry assays revealed that PTTG3P affected cell apoptosis. (**G**) Tumor volume and (**H**) weight were measured *in vivo* when injected with overexpressed PTTG3P in HCT116 cells. (**I**) CCK8 assay detected the proliferation of HT-29 cells transfected with overexpressed PTTG3P and treated with 2.5 mM 2-DG or 100 μM 3-BP. (**J**) Xenograft tumors volume, (**K**) xenograft tumors weight were established, with injected with PTTG3P or PTTG3P plus sh-LDHA or PTTG3P treated with 2-DG (1000 mg/kg, injected into the abdominal cavity). Empty vector as indicated. (**L**) Tumor volume and (**M**) weight were measured *in vivo* when injected with sh-PTTG3P (20 nmol twice per week) and Oxaliplatin treatment (5 mg/kg twice per week, injected into the abdominal cavity) transfected HCT116 cells. Data are presented as the mean ± SD from three independent experiments. *P<0.05, **P<0.01.

Next, we performed the glucose uptake analysis, ATP analysis, lactate production analysis, and discovered that sh-PTTG3P repressed these phenomena. In contrast, PTTG3P overexpression boosted glucose uptake (Figure 3A), lactate production (Figure 3B), and ATP accumulation (Figure 3C). Additionally, we calculated the level of ECAR, sh-PTTG3P notably repressed glycolytic capacity and *vice versa* (Figure 3D). Also, we found that silenced PTTG3P suppressed the proliferation, facilitated apoptosis of HCT116 cells, whereas up-regulated PTTG3P increased the proliferation, inhibited apoptosis of HT-29 cells according to the CCK-8 assay and flow cytometry analysis (Figure 3E,F). *In vivo*, highly expressed PTTG3P efficiently increased the tumor growth (Figure 3G,H). We then explored whether glycolysis played a vital role in cell proliferation and tumor growth. Notably, the glycolic inhibitors 2-DG and 3-BP or depletion of LDHA, which catalyzed the final step of glycolysis, could partly abrogate cancer cell proliferation and tumor growth (Figure 3I–K). Clinically, Oxaliplatin is used for the treatment of CRC. Previously, it is reported that suppression of glycolysis is an effective strategy to block cell proliferation and conquer drug resistance. As shown in Figure 3L,M, PTTG3P depletion and Oxaliplatin played a synergistic role in emancipating tumor growth. As a taken, PTTG3P knockdown plus Oxaliplatin is a promising therapy for CRC.

### PTTG3P regulates Hippo signaling pathway in CRC

**Figure 4:**
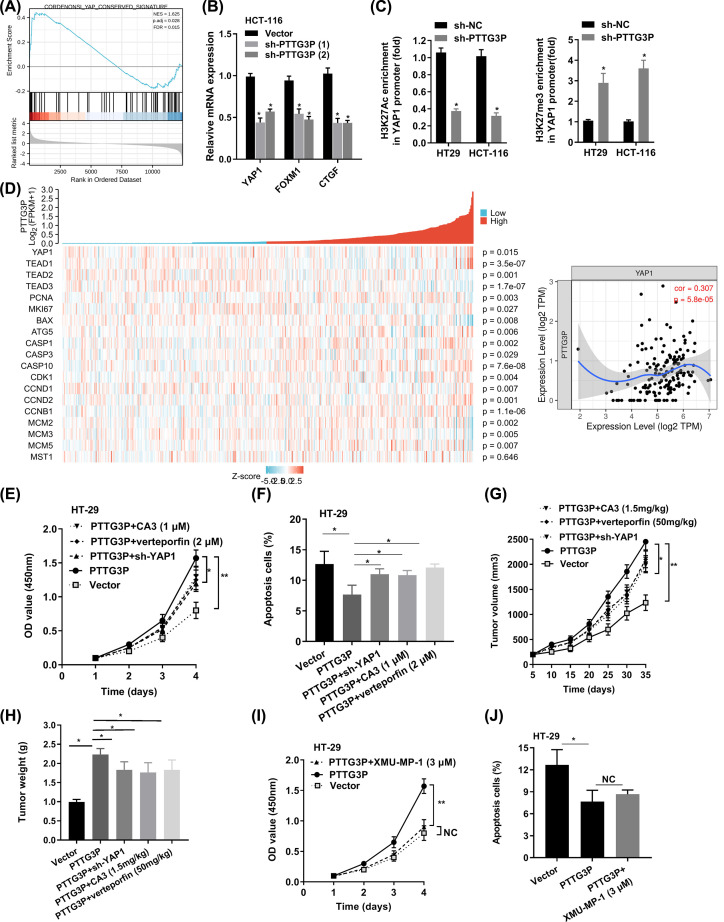
PTTG3P regulates Hippo signaling pathway in CRC (**A**) GSEA plot showing that PTTG3P expression positively correlated with YAP-activated gene signatures. (**B**) PTTG3P knockdown impaired the mRNA level of YAP1, FOXM1, and CTGF. (**C**) PTTG3P knockdown impaired the enrichment of H3K27Ac, H3K27me3 on the promoter of YAP1. (**D**) PTTG3P co-expression heat map, TCGA (https://portal.gdc.cancer.gov/) COAD, level 3 HTSeq-FPKM. (**E,F**) The effect of HT-29 cells transfected with PTTG3P or PTTG3P+sh-YAP1 or PTTG3P+CA3 (1 μM) or PTTG3P+verteporfin (2 μM) on cell proliferation by CCK8 assay and apoptosis by flow cytometry assays. (**G,H**) Xenograft tumor volume, xenograft tumor weight were established, with injected with PTTG3P or PTTG3P+sh-YAP1or PTTG3P+CA3 (1.5 mg/kg) or PTTG3P+verteporfin (50 mg/kg). (**I**) CCK8 assay detected the proliferation of HT-29 cells transfected with PTTG3P or PTTG3P plus XMU-MP-1 (3 μM). (**J**) Flow cytometry assays revealed that PTTG3P plus XMU-MP-1 (3 μM) could barely rescue cell apoptosis. Data are presented as the mean ± SD from three independent experiments. *P<0.05, **P<0.01.

In order to elucidate which pathway is involved in PTTG3P-mediated CRC progression, GSEA in the published TCGA CRC database was explored. And we suggested that PTTG3P expression was associated with the YAP1-activated gene signatures, indicating that Hippo signaling pathway might be involved (Figure 4A). Then the hub genes in the Hippo pathway, including LATS1/2, MST1/2 and YAP1, and Hippo pathway target genes, such as CDX2, FOXM1, CTGF and CYR61, were checked in sh-PTTG3P HCT116 cells. Subsequently, PTTG3P knockdown impaired the level of YAP1, FOXM1 and CTGF (Figure 4B). Moreover, silenced PTTG3P obviously decreased the enrichment of H3K27Ac at the YAP1 promoter, while that of H3K27me3 was increased (Figure 4C). Next, we explored the TCGA database and drew the PTTG3P co-expression heat map. Then, we discovered that PTTG3P expression was correlated with genes in the Hippo pathway (YAP1, TEAD1-3), genes in the phenotype of proliferation (PCNA, MKI67, MCM2, MCM3, MCM5), gene in the phenotype of apoptosis (BAX, CASP1, CASP3, CASP10), gene in the phenotype of autophagy (ATG5), and genes in the phenotype of cell cycle (CDK1, CCDN1, CCND2, CCNB1), but not with MST1, an upstream factor of YAP1 (Figure 4D).

It is commonly acknowledged that YAP1, a crucial factor in the Hippo pathway, involves in cell proliferation and suppresses apoptotic genes, and YAP1 was highly expressed in CRC (Supplementary Figure S1H,I), and associated with advanced characteristics of CRC (Supplementary Table S4). Further, YAP1 had a higher diagnostic value (AUC = 0.793, 95% CI: 0.729–0.858) from the TCGA database (Supplementary Figure S1J). Additionally, we performed rescue assays in HT-29 cells. PTTG3P OE plus YAP1 KD could reverse the bioeffect of PTTG3P. Besides, we applied YAP1 inhibitors, CA3 (a novel specific YAP1 inhibitor) and verteporfin (an inhibitor of YAP1/TEAD interaction), got the same conclusion (Figure 4E–H). Intriguingly, the treatment of Hippo pathway inhibitor, XMU-MP-1 (inhibiting MST1/2), could not recover the effect of PTTG3P on proliferation, apoptosis, and tumor growth (Figure 4I,J). In brief, all the data uncovered that PTTG3P hedges the key factor MST1/2, while modulates YAP1 in the Hippo pathway to exhibit pivotal functions in CRC progression.

### PTTG3P promotes M2 phenotype polarization of macrophage

Tumor microenvironment (TME) is composed of tumor cells and surrounding non-tumor stromal cells, mainly including tumor-associated macrophages (TAMs), endothelial cells, and carcinoma-associated fibroblasts (CAFs) [11,12]. With the occurrence of tumors, tumor cells secrete various chemokines to recruit monocytes to infiltrate tumor tissues and further promote their M2-type polarization. Importantly, M2-like TAMs can in turn accelerate tumor growth, promote tumor cell invasion and metastasis, and inhibit immune killing to promote tumor progression [13].

To elucidate whether PTTG3P play a role in M2 polarization, we explored the expression of PTTG3P, M1 markers (CD80, MCP-1, iNOS, and IL-6) and M2 markers (CD206 and MRC-2) in unpolarized macrophages, M1 macrophages treated by LPS/INF-γ and M2 macrophages treated by IL4/IL13.

**Figure 5:**
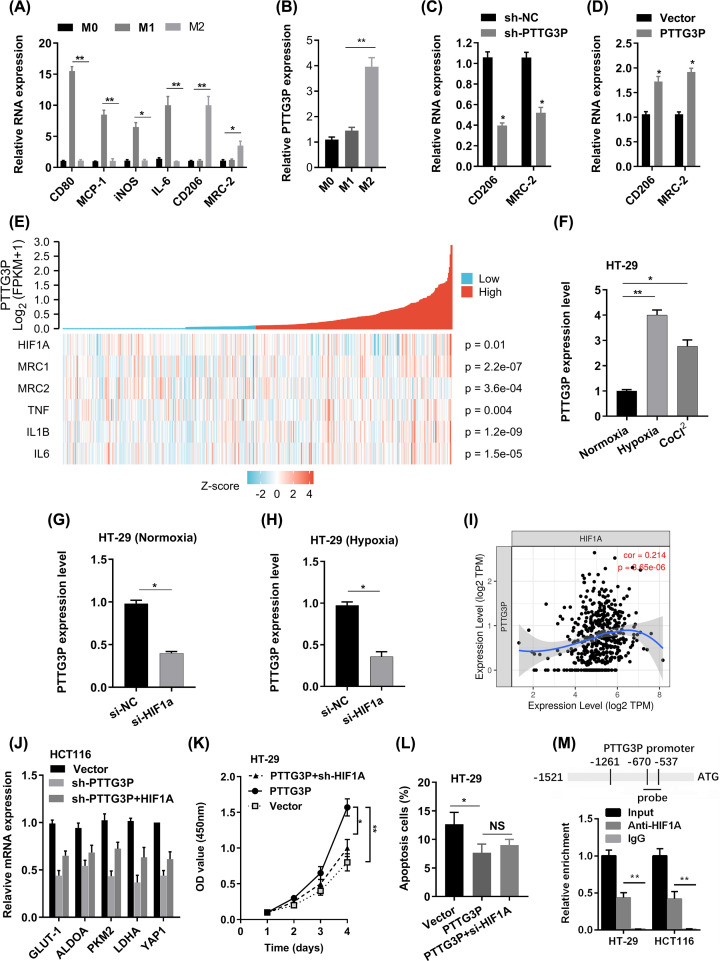
PTTG3P could induce M2 polarization of macrophage (**A**) RT-PCR was used to detect the expression of M1 markers and M2 markers after LPS/INF-γ or IL-4/IL-13 treatment. (**B**) The expression of PTTG3P was increased in M2 macrophages. (**C**) M2 markers (CD206 and MRC-2) were ablated in the sh-PTTG3P group. (**D**) M2 markers (CD206 and MRC-2) were increased in the PTTG3P group. (**E**) PTTG3P co-expression heat map, TCGA (https://portal.gdc.cancer.gov/) COAD, level 3 HTSeq-FPKM. (**F**)The expression of PTTG3P in HT-29 cells was measured after culturing under normoxia, hypoxia (1% O_2_), or CoCl_2_ (100 μM) for 24 h by qRT-PCR. (**G**) The expression of PTTG3P was evaluated by qRT-PCR in HT-29 cells after knockdown of HIF1A under normoxia condition. (**H**) The expression of PTTG3P was evaluated by qRT-PCR in HT-29 cells after knockdown of HIF1A under hypoxia condition. (**I**) PTTG3P correlated with HIF1A. (**J**–**L**) The effect of HT-29 cells transfected with PTTG3P or PTTG3P+si-HIF1A on downstream gene expression, cell proliferation, and apoptosis. (**M**) ChIP assay was performed to show HIF1A could directly bind to the PTTG3P promoter region *P<0.05, **P<0.01.

Our data indicated that the expression of PTTG3P, CD206 and MRC-2 considerably were elevated in M2 macrophages, and then CD80, MCP-1, iNOS and IL-6 were increased in M1 macrophages (Figure 5A), but PTTG3P was not increased in M1 macrophages (Figure 5B). Next, we knocked down PTTG3P with siRNA after propidium monoazide (PMA) treatment for 24 h, and utilized IL-4/ IL-13 to cause M2 phenotype. Interestingly, we found M2 markers (CD206 and MRC-2) were markedly diminished in si-PTTG3P group compared with si-NC group (Figure 5C), while enforced expression of PTTG3P did the opposite (Figure 5D). In addition, PTTG3P was correlated with CD206 (MRC1) and MRC2 from the TCGA-COAD database (Figure 5E).

### PTTG3P is regulated by HIF1A under hypoxic conditions

We next investigated the upstream factors which induced the elevated level of PTTG3P in CRC cells.

Then, we treated CRC cells with hypoxia or CoCl_2_ (hypoxia chemical inducer) and found the PTTG3P expression in HT-29 cells was obviously increased as well as the elevation of HIF1A (Figure 5F). While, depletion of HIF1A strikingly ameliorated PTTG3P expression in both normoxia and hypoxia conditions (Figure 5G,H). We also indicated that HIF1A and PTTG3P had a positive correlation (Figure 5E,I). HIF1A was associated with advanced characteristics of CRC from the TCGA-COAD database (Supplementary Table S5). Besides, HIF1A could partly rescue the effect of PTTG3P KD (Figure 5J–L). In addition, ChIP analysis indicated that HIF1A enriched in the PTTG3P promoter region (Figure 5M). Thus, the HIF1A/PTTG3P/YAP1 axis played a crucial role in CRC progression.

### PTTG3P plays vital functions in CRC immunology

**Figure 6:**
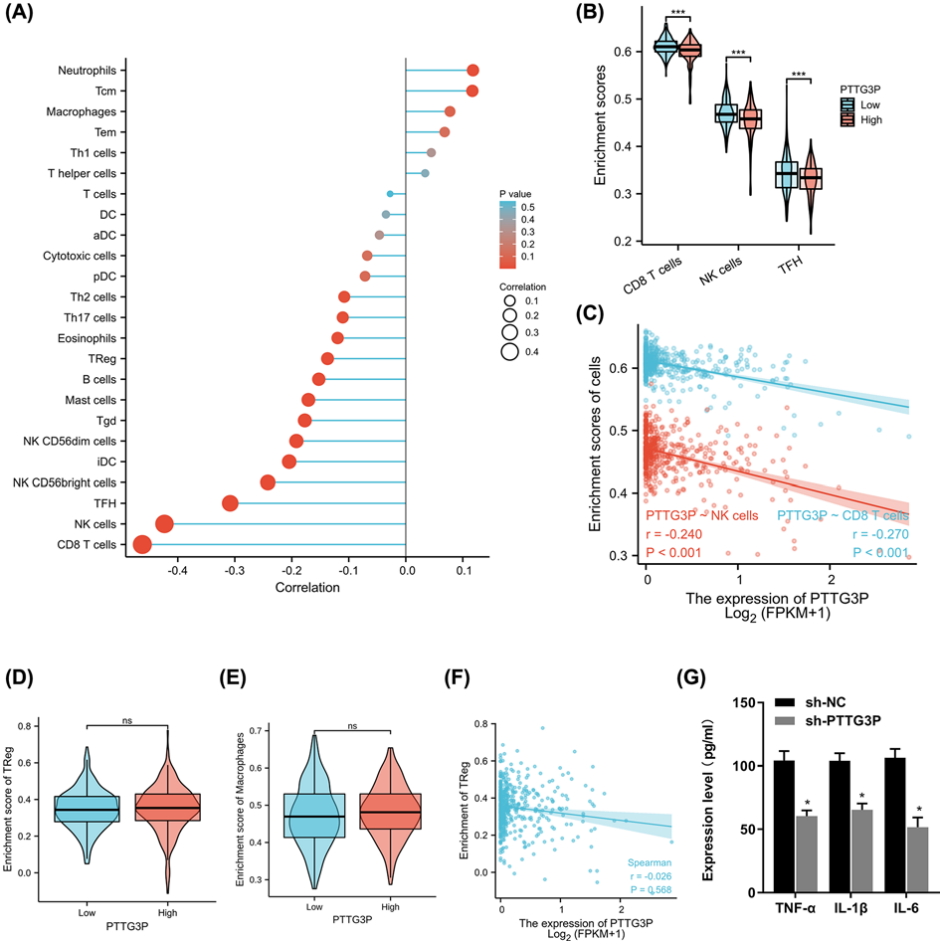
PTTG3P regulate in CRC immunology (**A**) The correlation of PTTG3P and immune cell correlation in TCGA-COAD database. (**B**) High and low PTTG3P expression with CD8^+^ T, NK, and TFH cells infiltration in TCGA-COAD database. (**C**) Scatter plot of PTTG3P expression and CD8^+^ T and NK cells infiltration in TCGA-COAD database. (**D,E**) High and low PTTG3P expression with Treg or macrophages infiltration in TCGA-COAD database. (**F**) Scatter plot of PTTG3P expression and Treg cells infiltration in TCGA-COAD database. (**G**) ELISA detection of the expression of TNF-α, IL-1β, and IL-6 in cell culture supernatants after depletion of PTTG3P in HCT116 cell.

Cancer cells have high glucose uptake and glycolysis, resulting in a low level of glucose in the tumor, thus inhibiting the production of IFN-r by CD8^+^ T cells in the tumor. The tumor immune environment broadly participates in different malignant tumors, including CRC. Recently, the treatment of CRC with immune checkpoint inhibitors (ICIs) has provided a potential clinical treatment. Interestingly, our findings proposed that low PTTG3P expression relates with CD8^+^ T, NK and TFH cells infiltration in the microenvironment of CRC, not with Treg or macrophages infiltration, based on the TCGA database (Figure 6A–F). And the results of ELISA showed that the level of inflammatory cytokines TNF-α, IL-1β and IL-6 were decreased with PTTG3P depletion (Figure 6G). In addition, PTTG3P was correlated with TNF-α, IL-1β and IL-6 from the TCGA-COAD database (Figure 5E).

## Discussion

Pseudogenes are nonfunctional segments of DNA that resemble functional genes. Most arise as superfluous copies of functional genes, either directly by DNA duplication or indirectly by reverse transcription of an mRNA transcript. For instance, lncRNA HK2P1 increased the lactate production and glucose uptake in endometrial stromal cells [14]. lncRNA PTENP1 suppressed the PI3K/AKT signaling pathway and hindered the progression of HCC [15]. However, the role of PTTG3P in regulating CRC glycolysis has not been fully elucidated. Our study uncovered that PTTG3P boosted cell proliferation and glycolysis via the miR-1271-5p/PTTG3P/YAP1 axis.

Our study verified that PTTG3P is highly expressed and has a potential diagnostic value, with an AUC of 0.776 (95% CI: 0.733–0.819) in CRC. Clinically, high PTTG3P expression considerably associates with tumor size and TNM stage as well as shorter survival time. These results confirmed that PTTG3P serves as a valuable prognostic biomarker and aids innovatively efficient therapies for CRC patients. Additionally, our findings stand in line with other research, Liu et al. [9] reported that PTTG3P was remarkably up-regulated in CRC tumor samples than that in normal samples. Zhou et al. [16] revealed that PTTG3P is a valuable resource for identification in HCC progression and is useful for biomarker development. Weng et al. [17] certified that PTTG3P facilitates cell proliferation, migration and invasion, and might serve as a new promising strategy for gastric cancer. Recently, PTTG3P expression has a relationship with breast cancer [18] and pancreatic cancer [19]. Thus, the oncogenic role of PTTG3P in malignant tumors is strongly suggested.

Malignant tumors could undergo glycolysis at a higher speed than non-tumor tissue controls [20–22]. This phenomenon is known as the Warburg effect [23]. The Warburg hypothesis demonstrates that malignant tumor is fundamentally caused by mitochondrial metabolism disorder. Doherty et al. [22] found that tumor lactate levels correlate with increased metastasis, tumor recurrence, and poor outcome. And targeting lactate metabolism is a prospective method for cancer therapeutics. Furthermore, cancer cells with a high level of glycolysis and acid resistance have an energetic growth advantage, which facilitates unrestrained proliferation and invasion. In our study, we explored gain- and loss-of-function approaches in HT-29 and HCT116 cells and found PTTG3P ablation resulted in the inhibition of CRC cell glycolysis by regulating numerous genes linked with metabolic pathways, whereas the opposite outcome was observed after enforced expression of PTTG3P. Nowadays, a ketogenic diet was used to constrain glycolysis to starve cancer cells, adjusting mitochondrial metabolism [24]. Here, we also proposed that the biological mechanism of PTTG3P on boosting cell proliferation might resist apoptosis.

Hippo signaling pathway has become increasingly important in human cancer [25], the key regulator YAP1 has been certified to be up-regulated in breast cancer, CRC and liver cancer [26], and YAP1 could promote cell growth [27–29] and inhibit apoptosis [30]. Clinically, YAP1 could be a target for the development of cancer drugs [31]. Yi et al. [32] suggested that inhibiting TEAD-YAP1 interactions or block the binding function of WW domains is a pharmacologically viable strategy against the YAP1 oncoprotein. In our presented study, we discovered that PTTG3P activates Hippo signaling pathway by promoting YPA1, FOXM1 and CTGF, not MST1/2, and rescue assay consolidates this by using the Hippo pathway inhibitor, XMU-MP-1 (inhibiting MST1/2).

These years, cancer has been considered to be a complex system including the TME. TAMs are the most common immune-related stromal cells in the TME, and communication between cancer cells and TAMs is crucial for the progression of epithelial ovarian cancer (EOC) [33]. Recently, a great amount of studies elucidated that TAMs has the property of M2 macrophages that are related to cancer progression. Our finding suggested that PTTG3P expression was elevated in M2 macrophages, not in M1 macrophages or unpolarized macrophages. Next, enforced PTTG3P markedly increased the M2 macrophages markers (CD206 and MRC-2), while PTTG3P depletion attenuated these markers. Hence, PTTG3P might play a vital role in M2 polarization progression.

During cancer progression, tumor cells acquire comprehensive metabolic reprogramming, and tissue hypoxia is a prominent feature of solid tumors leading to cell metabolism adaptive changes. HIF1A is a key oxygen-regulated transcriptional activator, playing a fundamental role in the adaptation of tumor cells to hypoxia by up-regulating the transcription of target genes related to multiple biological processes, including cell survival, proliferation, angiogenesis and anti-apoptosis [34,35]. In the microenvironment of oxygen-glucose shortage, HIF1A is a major factor in cancer survival. Zhou et al. [36] proposed that HIF1A activated lncRNA RAET1K modulated glycolysis in hepatocellular carcinoma cells via miR-100-5p. Tiwari et al. [37] discovered that HIF1A might act as a tumor suppressor by preventing the expression of PPP1R1B and subsequent degradation of the p53 protein in pancreatic cancer cells. Our study indicated that HIF1A increased PTTG3P expression at the transcription level and enriched in the promoter region of PTTG3P, and could partly rescue the bioeffect of PTTG3P. Last, our findings proposed that low PTTG3P expression relates with CD8^+^ T, NK and TFH cells infiltration in the microenvironment of CRC, not with Treg or macrophages infiltration from the TCGA database. And we found that a decreased TNF-α, IL-1β and IL-6 level was accompanied by ablation of PTTG3P.

In summary, we found hypoxia-induced PTTG3P was an oncogene in CRC, and higher PTTG3P expression was related to a dismal prognosis. PTTG3P might facilitate cell proliferation, glycolysis and M2 phenotype of macrophage. Thus, PTTG3P plus HIF1A should be together adopted as critical targets for the prevention and therapy of CRC, illuminating some light on the understanding of the lncRNAs in CRC progression.

## Supplementary Material

Supplementary Figures S1-S2 and Tables S1-S5

## Data Availability

The datasets used and analyzed in the current study are available from the corresponding authors on reasonable request.

## Abbreviations

ATP: adenosine triphosphate
AUC: area under the ROC curve
CCK8: Cell Counting Kit-8
ChIP: chromatin immunoprecipitation
CRC: colorectal cancer
ECAR: extracellular acidification rate
GSEA: gene set enrichment analysis
HIF1A: hypoxia-inducible factor-1α
LDHA3: Lactate dehydrogenase A3
lncRNA: long noncoding RNA
PTTG3P: pituitary tumor-transforming 3, pseudogene
ROC: receiver operating characteristic curve
TAM: tumor-associated macrophage
TCGA: The Cancer Genome Atlas
TFH: Follicular helper T cell
TME: tumor microenvironment
2-DG: 2-deoxyglucose
3-BG: 3-Butylene glycol
95% CI: 95% confidence interval
