# Supplementary material for: Hypoxia-induced PTTG3P contributes to colorectal cancer glycolysis and M2 phenotype of macrophage
Source: Biosci Rep. 2021 Jul 6;41(7):BSR20210764. doi: 10.1042/BSR20210764 (PMC8264182; doi:10.1042/BSR20210764)
Supplement: Supplementary Figures S1-S2 and Tables S1-S5 [file BSR-2021-0764_supp.pdf]

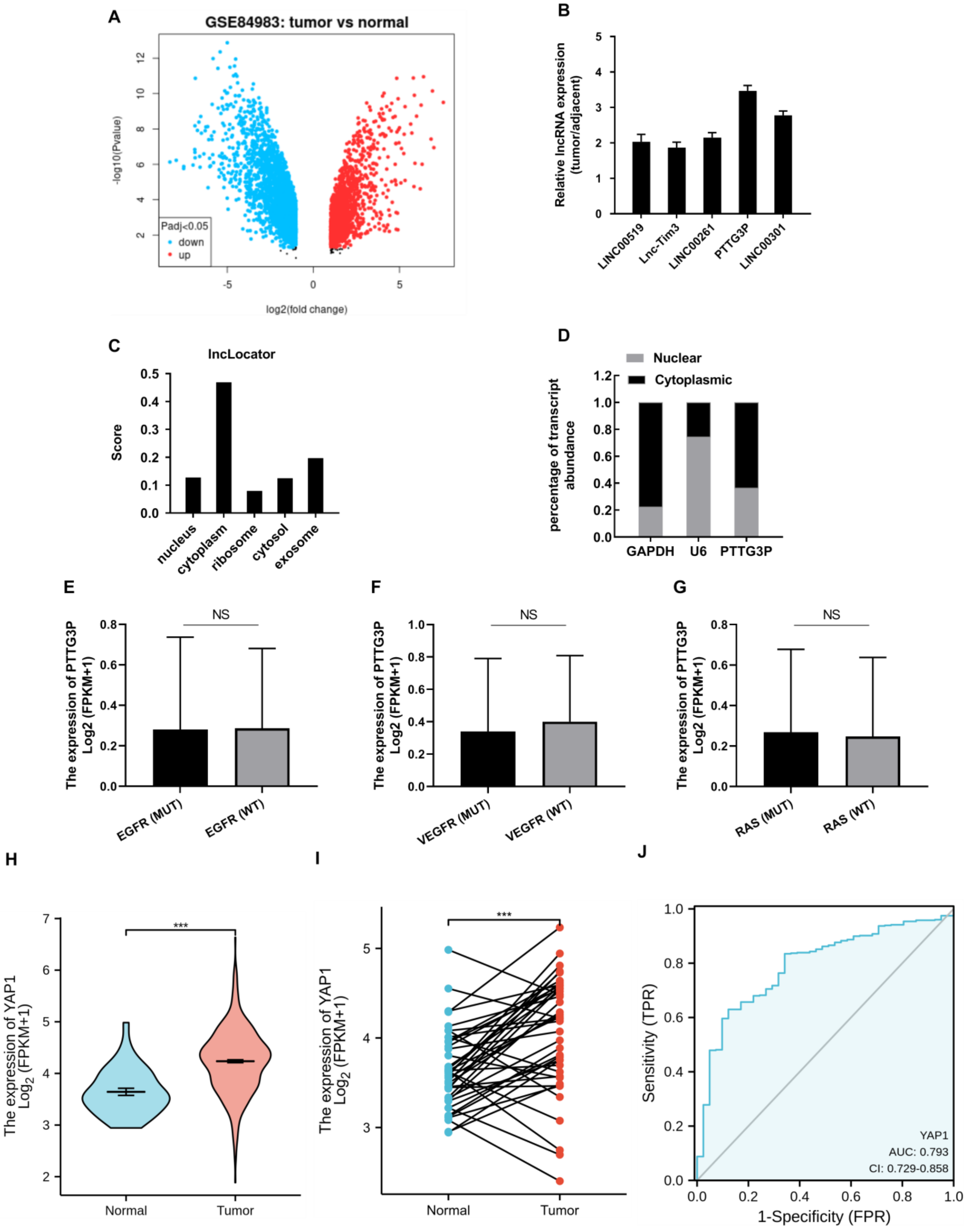
A
GSE84983: tumor vs normal
-log10(Pvalue)
log2(fold change)
Padj<0.05
down
up
B
Relative lncRNA expression (tumor/adjacent)
LINC00519
Lnc-Tim3
LINC00261
PTTG3P
LINC00301
C
lncLocator
Score
nucleus
cytoplasm
ribosome
cytosol
exosome
D
Nuclear
Cytoplasmic
percentage of transcript abundance
GAPDH
U6
PTTG3P
E
F
G
The expression of PTTG3P Log2 (FPKM+1)
NS
EGFR (MUT)
EGFR (WT)
VEGFR (MUT)
VEGFR (WT)
RAS (MUT)
RAS (WT)
H
I
J
The expression of YAP1 Log_2 (FPKM+1)
***
Normal
Tumor
Sensitivity (TPR)
1-Specificity (FPR)
YAP1
AUC: 0.793
CI: 0.729-0.858

Figure S1 The selection of PTTG3P and PTTg3P, YAP1 expression

(A) According to an online database (GSE84983), the volcano graph showed differential gene.

(B) qRT-PCR of the expressions of the top 5 up-regulated lncRNAs in CRC.

(C) PTTG3P localization was predicted using lncLocator (lncRNA subcellular localization predictor).

(D) PTTG3P localization was validated by subcellular fractionation.

(E-G)Difference in the expression of PTTG3P between CRC samples with somatic mutations in EGFR, VEGFR or RAS and those without such mutations.

(H, I) High YAP1 expression was observed in CRC (TCGA-COAD, n = 521).

(J) ROC curve analysis of the diagnostic value of YAP1.

*P < 0.05, **P < 0.01, ***P < 0.001

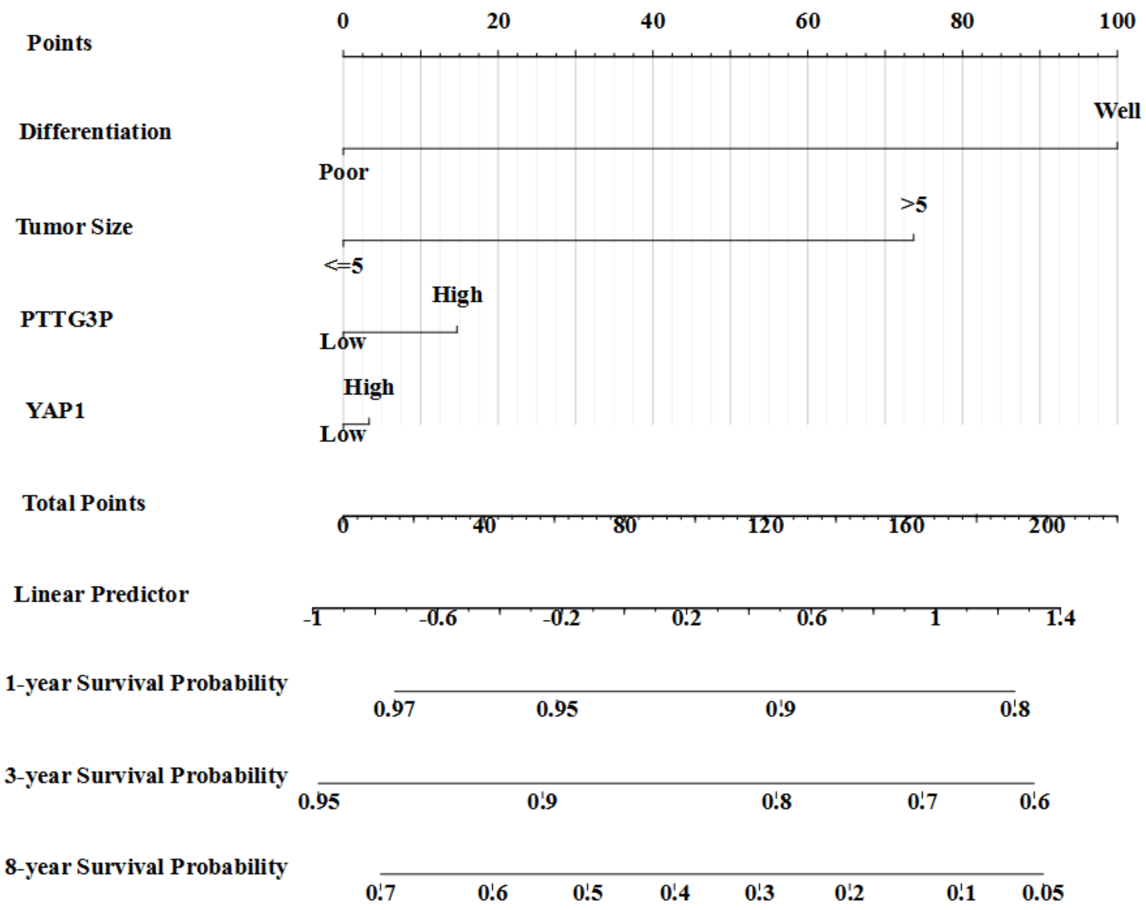
Points
0
20
40
60
80
100
Differentiation
Well
Poor
Tumor Size
>5
<=5
PTTG3P
High
Low
YAP1
High
Low
Total Points
0
40
80
120
160
200
Linear Predictor
-1
-0.6
-0.2
0.2
0.6
1
1.4
1-year Survival Probability
0.97
0.95
0.9
0.8
3-year Survival Probability
0.95
0.9
0.8
0.7
0.6
8-year Survival Probability
0.7
0.6
0.5
0.4
0.3
0.2
0.1
0.05

Figure S2 Radiomics nomogram

Developed radiomics nomogram, the radiomics nomogram was developed with tumor differentiation, tumor size, PTTG3p expression, and YAP1 expression incorporated.

Supplementary Table 1. sh-RNA sequences.

shRNA	Target sequence
sh-PTTG3P(1)	GGTTGAGAGCGGCAATAATCC
sh-PTTG3P(2)	GCATCCTTGTGGCTACAAAGG
sh-LDHA	GTTCACAAGCAGGTGGTTGAGAGTGCTTA
sh-PGK1	ACAACCAGAGGATTAAGGC
sh-YAP1(1)	GGUCAGAGAUACUUCUUAAAU
sh-YAP1(2)	GGUGAUACUAUCAACCAAAGC

Supplementary Table 2. All primers for real-time PCR assay

Primer	Former	Reverse
PTTG3P	GGGGTCTGGACCTTCAATCAA	GCTTTAGGTAAGGATGTGGGA
miR-1271-5p	CAGCACTTGGCACCTAGCA	TATGGTTGTTCTCCTCTCTGTCTC
YAP1	TACGATACAAGGCTGTTAGAGAG	TTGAGATGCATGCTTTGCATAC
LDHA	ATGGCAACTCTAAAGGATCA	GCAACTTGCAGTTCGGGC
GLUT-1	GGCCAAGAGTGTGCTAAAGAA	ACAGCGTTGATGCCAGACAG
PKM2	ATGTCGAAGCCCCATAGTGAA	TGGGTGGTGAATCAATGTCCA
ALDOA	AGGCCATGCTTGCACTCAGAAGT	AGGGCCCAGGGCTTCAGCAGG
CTGF	CAGCATGGACGTTCGTCTG	CAGCATGGACGTTCGTCTG
CYR61	CTCGCCTTAGTCGTCACCC	CTCGCCTTAGTCGTCACCC
U6	GATTTCTCCCTCATCGCTTACAG	CTGCTTCATGATCGTTGTTGCTTG
GAPDH	GGGAGCCAAAAGGGTCATCA	TGATGGCATGGACTGTGGTC

Supplementary Table 3. Protein coding potential

Metric	Raw result	interpretation
PRIDE reprocessing 2.0	0	Non-coding
Lee translation initiation sites	0	Non-coding
PhyloCSF score	<0	Non-coding
CPAT coding probability	2.33%	Non-coding
Bazzini small ORFs	0	Non-coding

Supplementary Table 4. Correlation between YAP1 expression and clinicopathologic characteristics of CRC patients

Characteristic	Low expression of YAP1	High expression of YAP1	p
n	239	239	
T stage, n (%)			0.756
T1	7 (1.5%)	4 (0.8%)	
T2	39 (8.2%)	44 (9.2%)	
T3	161 (33.8%)	162 (34%)	
T4	31 (6.5%)	29 (6.1%)	
N stage, n (%)			0.235
N0	151 (31.6%)	133 (27.8%)	
N1	50 (10.5%)	58 (12.1%)	
N2	38 (7.9%)	48 (10%)	
M stage, n (%)			0.046
M0	182 (43.9%)	167 (40.2%)	
M1	25 (6%)	41 (9.9%)	
Pathologic stage, n (%)			0.086
Stage I	40 (8.6%)	41 (8.8%)	
Stage II	104 (22.3%)	83 (17.8%)	
Stage III	63 (13.5%)	70 (15%)	
Stage IV	25 (5.4%)	41 (8.8%)	
Primary therapy outcome, n (%)			0.466
PD	9 (3.6%)	16 (6.4%)	
SD	1 (0.4%)	3 (1.2%)	
PR	6 (2.4%)	7 (2.8%)	
CR	104 (41.6%)	104 (41.6%)	
Gender, n (%)			0.169
Female	121 (25.3%)	105 (22%)	
Male	118 (24.7%)	134 (28%)	
Race, n (%)			0.457
Asian	4 (1.3%)	7 (2.3%)	
Black or African American	23 (7.5%)	40 (13.1%)	
White	104 (34%)	128 (41.8%)	
Weight, n (%)			0.768
<=90	84 (30.8%)	105 (38.5%)	
>90	35 (12.8%)	49 (17.9%)	
Age, n (%)			0.226
<=65	90 (18.8%)	104 (21.8%)	

>65	149 (31.2%)	135 (28.2%)	
Height, n (%)			0.134
<170	61 (23.8%)	66 (25.8%)	
>=170	49 (19.1%)	80 (31.2%)	
BMI, n (%)			0.814
<25	36 (14.1%)	51 (19.9%)	
>=25	74 (28.9%)	95 (37.1%)	
Residual tumor, n (%)			0.430
R0	185 (49.5%)	161 (43%)	
R1	1 (0.3%)	3 (0.8%)	
R2	11 (2.9%)	13 (3.5%)	
CEA level, n (%)			0.404
<=5	101 (33.3%)	95 (31.4%)	
>5	49 (16.2%)	58 (19.1%)	
Perineural invasion, n (%)			0.329
NO	57 (31.5%)	78 (43.1%)	
YES	15 (8.3%)	31 (17.1%)	
Lymphatic invasion, n (%)			0.569
NO	129 (29.7%)	137 (31.6%)	
YES	87 (20%)	81 (18.7%)	
History of colon polyps, n (%)			0.038
NO	123 (30.1%)	139 (34.1%)	
YES	85 (20.8%)	61 (15%)	
Colon polyps present, n (%)			0.965
NO	67 (26.9%)	95 (38.2%)	
YES	37 (14.9%)	50 (20.1%)	
Neoplasm type, n (%)			1.000
Colon adenocarcinoma	239 (50%)	239 (50%)	
Rectum adenocarcinoma	0 (0%)	0 (0%)	
OS event, n (%)			0.373
Alive	192 (40.2%)	183 (38.3%)	
Dead	47 (9.8%)	56 (11.7%)	
DSS event, n (%)			0.385
Alive	201 (43.5%)	197 (42.6%)	

Dead	28 (6.1%)	36 (7.8%)	
PFI event, n (%)			0.121
Alive	183 (38.3%)	167 (34.9%)	
Dead	56 (11.7%)	72 (15.1%)	
Age, meidan (IQR)	69 (60, 77)	68 (57, 77)	0.287

Supplementary Table 5. Correlation between HIF1A expression and clinicopathologic characteristics of CRC patients

Characteristic	Low expression of HIF1A	High expression of HIF1A	p
n	239	239	
T stage, n (%)			0.380
T1	7 (1.5%)	4 (0.8%)	
T2	45 (9.4%)	38 (8%)	
T3	162 (34%)	161 (33.8%)	
T4	25 (5.2%)	35 (7.3%)	
N stage, n (%)			0.647
N0	137 (28.7%)	147 (30.8%)	
N1	57 (11.9%)	51 (10.7%)	
N2	45 (9.4%)	41 (8.6%)	
M stage, n (%)			0.184
M0	172 (41.4%)	177 (42.7%)	
M1	39 (9.4%)	27 (6.5%)	
Pathologic stage, n (%)			0.253
Stage I	41 (8.8%)	40 (8.6%)	
Stage II	84 (18%)	103 (22.1%)	
Stage III	67 (14.3%)	66 (14.1%)	
Stage IV	39 (8.4%)	27 (5.8%)	
Primary therapy outcome, n (%)			0.698
PD	10 (4%)	15 (6%)	
SD	2 (0.8%)	2 (0.8%)	
PR	7 (2.8%)	6 (2.4%)	
CR	109 (43.6%)	99 (39.6%)	
Gender, n (%)			0.314
Female	107 (22.4%)	119 (24.9%)	
Male	132 (27.6%)	120 (25.1%)	

Race, n (%)			0.053
Asian	3 (1%)	8 (2.6%)	
Black or African American	35 (11.4%)	28 (9.2%)	
White	94 (30.7%)	138 (45.1%)	
Age, n (%)			1.000
<=65	97 (20.3%)	97 (20.3%)	
>65	142 (29.7%)	142 (29.7%)	
Weight, n (%)			0.592
<=90	84 (30.8%)	105 (38.5%)	
>90	41 (15%)	43 (15.8%)	
Height, n (%)			0.370
<170	52 (20.3%)	75 (29.3%)	
>=170	61 (23.8%)	68 (26.6%)	
BMI, n (%)			0.441
<25	35 (13.7%)	52 (20.3%)	
>=25	78 (30.5%)	91 (35.5%)	
Residual tumor, n (%)			0.071
R0	172 (46%)	174 (46.5%)	
R1	1 (0.3%)	3 (0.8%)	
R2	17 (4.5%)	7 (1.9%)	
CEA level, n (%)			0.601
<=5	95 (31.4%)	101 (33.3%)	
>5	56 (18.5%)	51 (16.8%)	
Perineural invasion, n (%)			0.044
NO	62 (34.3%)	73 (40.3%)	
YES	13 (7.2%)	33 (18.2%)	
Lymphatic invasion, n (%)			0.143
NO	122 (28.1%)	144 (33.2%)	
YES	90 (20.7%)	78 (18%)	
History of colon polyps, n (%)			0.526
NO	130 (31.9%)	132 (32.4%)	
YES	78 (19.1%)	68 (16.7%)	

Colon polyps present, n (%)			0.352
NO	65 (26.1%)	97 (39%)	
YES	41 (16.5%)	46 (18.5%)	
Neoplasm type, n (%)			1.000
Colon adenocarcinoma	239 (50%)	239 (50%)	
Rectum adenocarcinoma	0 (0%)	0 (0%)	
OS event, n (%)			0.373
Alive	183 (38.3%)	192 (40.2%)	
Dead	56 (11.7%)	47 (9.8%)	
DSS event, n (%)			0.390
Alive	191 (41.3%)	207 (44.8%)	
Dead	35 (7.6%)	29 (6.3%)	
PFI event, n (%)			0.757
Alive	173 (36.2%)	177 (37%)	
Dead	66 (13.8%)	62 (13%)	
Age, meidan (IQR)	69 (58, 75.5)	69 (59.5, 78)	0.471
